# Serological Levels of Anti-clathrin Antibodies Are Decreased in Patients With Pseudoexfoliation Glaucoma

**DOI:** 10.3389/fimmu.2021.616421

**Published:** 2021-02-19

**Authors:** Vanessa M. Beutgen, Norbert Pfeiffer, Franz H. Grus

**Affiliations:** Experimental and Translational Ophthalmology, Department of Ophthalmology, University Medical Center of the Johannes Gutenberg-University Mainz, Mainz, Germany

**Keywords:** autoantibodies, autoimmunity, glaucoma, microarray, bioinformatics, immunoproteomics

## Abstract

Evidence for immunologic contribution to glaucoma pathophysiology is steadily increasing in ophthalmic research. Particularly, an altered abundance of circulating autoantibodies to ocular antigens is frequently observed. Here, we report an analysis of autoantibody abundancies to selected antigens in sera of open-angle glaucoma patients, subdivided into normal-tension glaucoma (*N* = 31), primary open-angle glaucoma (*N* = 43) and pseudoexfoliation glaucoma (*N* = 45), vs. a non-glaucomatous control group (*N* = 46). Serum samples were analyzed by protein microarray, including 38 antigens. Differences in antibody levels were assessed by ANOVA. Five serological antibodies showed significantly altered levels among the four groups (*P* < 0.05), which can be used to cluster the subjects in groups consisting mainly of PEXG or POAG/NTG samples. Among the altered autoantibodies, anti-Clathrin antibodies were identified as most important subgroup predictors, enhancing prospective glaucoma subtype prediction. As a second aim, we wanted to gain further insights into the characteristics of previously identified glaucoma-related antigens and their role in glaucoma pathogenesis. To this end, we used the bioinformatics toolset of Metascape to construct protein-protein interaction networks and GO enrichment analysis. Glaucoma-related antigens were significantly enriched in 13 biological processes, including mRNA metabolism, protein folding, blood coagulation and apoptosis, proposing a link of glaucoma-associated pathways to changes in the autoantibody repertoire. In conclusion, our study provides new aspects of the involvement of natural autoimmunity in glaucoma pathomechanisms and promotes advanced opportunities toward new diagnostic approaches.

## Introduction

Glaucoma is a group of progressive neurodegenerative diseases of the optic nerve with variating forms of manifestation. An open-angle glaucoma (OAG) manifests as atrophy of the optic nerve and resulting vision loss while a normal iridiocorneal angle is maintained. The disease subtypes are assumed to have multifactorial pathogenesis, but few factors can be considered as significant hallmarks. Primary open-angle glaucoma (POAG), the most common form, is characterized by a typical optic nerve cupping, caused by the death of retinal ganglion cells (RGCs) and their axons, along with an increased intraocular pressure (IOP) that is mainly caused by dysregulations in the trabecular meshwork (TM) (1–3). An elevated IOP can also be observed in secondary glaucoma forms. In pseudoexfoliation syndrome (PEX), affected patients have a higher chance to develop pseudoexfoliation glaucoma (PEXG) (4). PEX is the most frequent reason for secondary open-angle glaucoma (5). Here, the high IOP is caused by an accumulation of pseudoexfoliation material in the anterior angle chamber, blocking the aqueous humor (AH) outflow in the TM (6). Another form of OAG that develops independently from increased IOP is normal-tension glaucoma (NTG), as reviewed in (7). Here, glaucomatous damage can be observed despite what is generally considered as physiological IOP (<21 mmHG). This implicates that disease mechanisms other than mere mechanical stress are likely to be involved in this subtype. One component adding to the possible disease mechanisms has been identified to be of an immunological kind. Signs of neuroinflammation and alterations in the innate immunity have been described in the context of glaucoma (8–12). One aspect here is also the disease-specific alteration of the natural autoantibody repertoire, as has been shown in different studies over the past years (13–16). Several alterations in specific serological autoantibodies targeting ocular antigens have been recently identified. Thus, we wanted to further investigate whether the identified autoantibodies also show specifically altered levels in different subtypes of glaucoma, as already indicated in earlier studies of our research group (17–19). To this end, we analyzed sera from POAG, NTG, and PEXG patients in comparison to a non-glaucomatous control group using protein microarray, also to identify potential markers that allow discrimination between OAG subtypes.

While the discovery of serological alterations of the autoantibody repertoire can be viable for diagnostic purposes when used as biomarkers, it tells us only little about their origin and function in the context of the disease. We wanted to look further into possible modes of action of autoantibodies that are altered in a disease specific manner. Thus, we also explored common characteristics of previously identified glaucoma-related antigens and investigate their relation to glaucoma pathogenesis. Autoantibodies to several ocular antigens have been frequently found by different groups using various methods, but they have not been regarded in a holistic approach. Most previous studies focus only on the identification of new markers but miss to put them in a context of disease mechanisms that allow new hypotheses to explain their role in OAG. To close this gap, we searched previously detected glaucoma-related antigens in the literature in addition to the one identified in this present study and analyzed their interactions and connections with the disease using bioinformatics tools.

## Results

### Analysis of Autoantibody Levels in Open-Angle Glaucoma Subtypes

**Figure 1:**
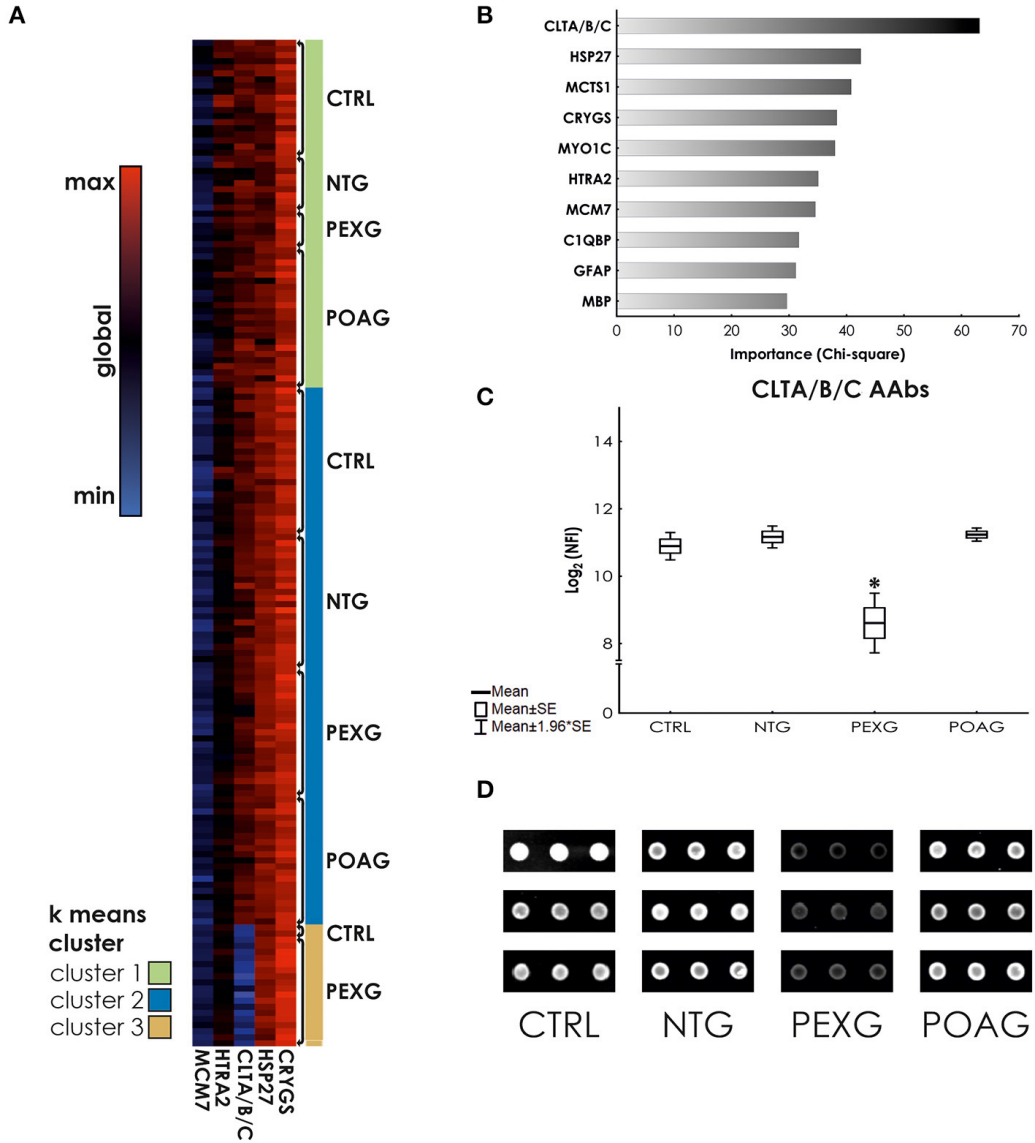
K means clustering and predictor screening. **(A)** Heat map showing autoantibody reactivities in glaucoma subtypes and control samples. **(B)** Predictor screening of autoantibody levels. The most important predictor for group affiliation are CLTA/B/C antibody levels. **(C)** ANOVA with HSD unequal N *post-hoc* test. CLTA/B/C autoantibody levels are significantly decreased in serum of PEXG patients compared to the other groups. **(D)** Representative examples of CLTA/B/C antibody reactivity on processed microarrays of individual samples. *Indicates statistical significance at *P* < 0.05.

In this study, we used a set of 38 antigens associated with glaucoma and other neurodegenerative diseases for the preparation of antigen microarrays (Array design in Supplementary File 1). We analyzed the IgG autoantibody reactivity to these antigens in glaucoma patients, comprising NTG (*N* = 31), POAG (*N* = 43), and PEXG (*N* = 45), and a non-glaucomatous control group (CTRL; *N* = 46). To identify alterations in antibody reactivity among these groups, we performed an ANOVA with consecutive *post-hoc* test (Tukey's HSD test for unequal N). Figure 1A shows a heat map for the five significantly (*P* < 0.05) altered autoantibody levels in either group. *Post-hoc* testing revealed significant differences as follows: HTRA2 antibodies show significantly decreased levels in PEXG compared to CTRL (*P* = 0.022). Autoantibodies to HSP27 and CRYGS are significantly increased in PEXG compared to CTRL (*P* < 0.001) and POAG (*P* = 0.001). PEXG patients show decreased levels of CLTA/B/C autoantibodies compared to all other groups (*P* < 0.001). Group differences were not significant in the *post-hoc* test for MCM7. These results are also available in Supplementary File 2. Measurements of autoantibody reactivity to ACTA1, ACTN1, ADRB2, ATP5A1, DDX46, ENO1, FN1, MECP2, PKM2, PROK, SOD, TGFB1I1, TTR, and VIM were not possible because the protein spots did not pass quality requirements (maximum of 30% missing values) or signals were below the detection limit.

Using *k* means clustering on all cases, three major clusters could be identified. Cluster 1 is mainly comprised (*X*^2^ *P* < 0.001) of POAG cases (40.35%), Cluster 2 allows no classification of groups (*X*^2^ *P* = 0.958), whereas Cluster 3 mostly contains PEXG cases (86.36%, *X*^2^ *P* < 0.001) (See also Table 1). To investigate, which autoantibody levels have the biggest impact on the clustering, we used a feature selection algorithm (Statistical Feature Selection and Variable Screening) to find the best predictors. We identified the antibodies to Clathrin (CLTA/BC) as the most important predictor (*X*^2^= 63.14; *P* < 0.01) (Figure 1B). ANOVA and Tukey's HSD *post-hoc* test revealed that Clathrin autoantibody levels are significantly decreased in the serum of PEXG patients compared to the other groups (Figure 1C). An exemplary representation of the arrays of these autoantibodies is also depicted in Figure 1D.

**Table 1:** Distribution of glaucoma cases and controls in the three *k* means clusters.

**%**	**CTRL**	**NTG**	**PEXG**	**POAG**	**Sum**
Cluster 1	33.33	15.79	10.53	40.35	100
Cluster 2	27.27	25	23.86	23.86	100
Cluster 3	10	0	90	0	100
**Cases**	27.27	18.79	27.27	26.67	100

*Cluster 1 mainly contains cases of the POAG group (40.35%). Cluster 2 is equally separated among the four groups. Cluster 3 predominantly contains PEXG cases (90%). X^2^-test is significant at P < 0.001 for cluster 1 and 3. Colors visualize values relative to the theoretical minimum 0% (blue) and to the theoretical maximum 100% (red). Blue indicates values below the expected value, red indicates values above the expected value. Light colors represent values close to the expected value*.

### Correlation of Autoantibodies With Clinical Features

Major hallmarks of glaucoma pathogenesis are IOP, visual field defects [measured as (“mean deviation” (MD)] and the cupping of the optic nerve that is assessed as the cup-disc ratio (CDR). We performed a Pearson correlation analysis to examine whether the serological antibody levels show an association with these clinical parameters (Table 2). The results reveal a weak negative correlation of CLTA/B/C autoantibodies with IOP (*r* = −0.26) and a weak positive correlation with CDR (*r* = 0.237). Further, a weak negative correlation of CRYGS autoantibodies (*r* = −0.27) with CDR was revealed.

**Table 2:** Pearson correlation analysis.

	**IOP (*N* = 160)**	**MD (*N* = 77)**	**CDR (*N* = 113)**
HTRA2	−0.088	−0.073	0.042
MCM7	−0.082	0.112	0.170
HSP27	0.077	−0.111	−0.175
CRYGS	0.024	−0.195	−0.270
CLTA/B/C	−0.255	0.037	0.237

*Table shows correlation coefficients (r). Marked entries (red) are significant at P < 0.05. Analysis was carried out with all available cases*.

### Bioinformatics Analysis of Established Glaucoma-Related Autoantigens

**Figure 2:**
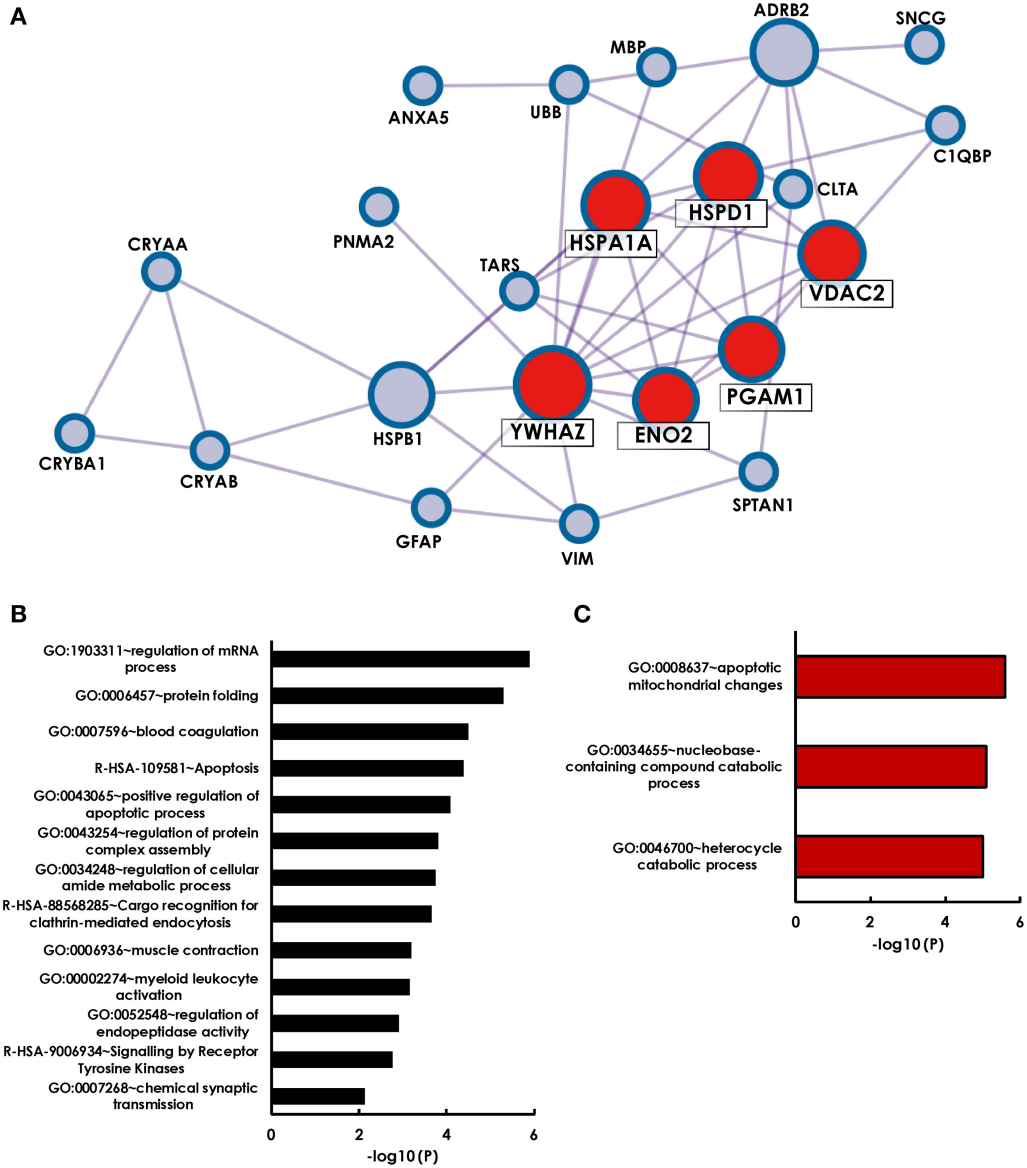
Holistic bioinformatics analysis of previously established glaucoma-related autoantigens. **(A)** Protein-protein interaction network. Twenty-two of the twenty-eight glaucoma-related antigens have at least one interaction partner among each other. Especially strong interactions were observed for six antigens (HSPA1A, HSPD1, YWHAZ, VDAC2, PGAM1, ENO2), that were identified by the Metaspcape algorithm MCODE (marked with red spots). **(B)** GO analysis of 28 glaucoma-related antigens. Shown are significantly enriched GO terms of biological processes and reactome gene sets. **(C)** GO analysis of MCODE cluster antigens only.

Starting with a literature search, we identified 28 antigens that have been identified as targets to glaucoma-related autoimmunity by different groups and methods (Table 3). To get an overall impression of the characteristics of theses previously identified glaucoma-related antigens, we conducted a protein-protein interaction analysis, as well as a GO term enrichment analysis using Metascape. We found that 22 of the 28 antigens had at least one interaction partner among the tested proteins (Figure 2A). A strong network was identified by Metascapes' Molecular Complex Detection algorithm (MCODE) with exceptionally high number of interactions for six of the antigens. These antigens are HSPA1A, HSPD1, YWHAZ, ENO2, PGAM1, and VDAC2. The GO enrichment analysis for all 28 antigens revealed 13 terms describing biological processes as significantly enriched. The most significantly enriched processes were regulation of mRNA process, protein folding, blood coagulation and apoptosis (Figure 2B). The GO enrichment analysis for the MCODE cluster of antigens only, revealed a significant enrichment of three biological processes. These comprise apoptotic mitochondrial changes, nucleobase-containing catabolic processes and heterocycle catabolic processes (Figure 2C). Regarding the cellular components of the MCODE cluster of antigens, three compartments were enriched (Table 4). Four of the six antigens are located in the myelin sheath and/or in mitochondria. Also, 50% of these antigens are to be found in the extracellular space.

**Table 3:** Glaucoma-related antigens identified in different previous studies.

**Protein**	**Gene ID**	**References**
HSP60	HSPD1	(20)
HSP27	HSPB1	(13, 21)
HSP70	HSPA1	(22)
Alpha A-crystallin	CRYAA	(21)
Alpha B-crystallin	CRYAB	(22)
β-L-crystallin	CRYBA1	(13)
Annexin 5	ANXA5	(13)
Ubiquitin	Isoform not specified; UBB used for analysis	(13)
Glial fibrillary acidic protein	GFAP	(13, 18)
14-3-3	Isoform not specified; YWHAZ used for analysis	(23)
Alpha fodrin	SPTAN1	(24)
Gamma enolase	ENO2	(25, 26)
Vimentin	VIM	(22)
Myelin basic protein	MBP	(13, 19)
Retinaldehyde-binding protein	RLBP1	(18)
Glutathion-S-transferase	GST	(27)
Retinal S-antigen	SAG	(18)
Histone H4	H4	(18)
Alpha 1 antitrypsin	SERPINA1	(13)
Gamma synuclein	SNCG	Claimed in (28)
Voltage-dependent anion-selective channel protein 2	VDAC2	(14)
Caldesmon	CALD1	(14)
Phosphoglycerate mutase 1	PGAM1	(14)
Threonine–tRNA ligase 1, cytoplasmic	TARS1	(16)
Complement component 1 Q subcomponent-binding protein, mitochondrial	C1QBP	(16)
Paraneoplastic antigen Ma2	PNMA2	(16)
Beta-2 adrenergic receptor	ADRB2	(29)
Clathrin	CLTA/B/C	This study

**Table 4:** Cellular components of MCODE clustered antigens.

**GO term**	**Cellular component**	**Count**	**%**	***P-*value**	**Antigens**	**List total**	**Pop hits**	**Pop total**	**Fold enrichment**
GO:0043209	Myelin sheath	4	66.67	<0.001	ENO2, PGAM1, HSPD1, VDAC2	6	152	18,224	79.930
GO:0005739	Mitochondrion	4	66.67	0.003	YWHAZ, HSPA1A, HSPD1, VDAC2	6	1,331	18,224	9.128
GO:0005615	Extracellular space	3	50	0.047	YWHAZ, ENO2, HSPD1	6	1,347	18,224	6.765

*DAVID GO enrichment analysis*.

## Discussion

In this study, we analyzed serological autoantibodies in sera of OAG patients and non-glaucomatous controls by means of an antigen microarray approach. We found altered levels of antibodies to CLTA/B/C, CRYGS, HSP27, and HTRA2 using ANOVA. Although the Pearson correlation analysis revealed few significant correlations between some autoantibody levels and glaucoma hallmarks, the relationships are very weak. With a correlation coefficient of |*r*| < 0.3 the correlations can be considered negligible. Thus, it can be concluded that the abundance of the analyzed antibodies is not essentially dependent on alterations of IOP, MD or CDR and *vice versa*. Although, some biological processes involved in pathological changes influencing IOP, and advancing neurodegeneration leading to increasing CDRs could have stronger dependencies leading to the observed statistical significance, but this cannot be deduced from the data at hand.

We analyzed, whether these autoantibodies might serve as disease markers to classify non-glaucomatous subjects and OAG subtypes. An earlier study of our research group already found significant differences between the antibody profiles of POAG and NTG, also in comparison to a control group, indicating their diagnostic value for OAG subtype discrimination (19). Another previous study showed significant differences between the antibody profiles to retinal antigens in aqueous humor of patients with PEXG and a control group (17). This study also compared the antibody profiles of POAG and PEXG patients. Although these two forms of OAG have vast differences in their pathogenesis, they showed no significant differences in their antibody profiles between each other. For a potential diagnostic discrimination of various glaucoma subtypes, suitable biomarker candidates need to be identified. Here, we achieved to find a putative autoantibody biomarker that shows significantly altered serological levels in PEXG compared to the other most common OAG types, POAG and NTG. Using a predictor screening algorithm, we identified antibodies to CLTA/B/C (Clathrin) as the most important predictor.

Clathrin is a protein majorly involved in the formation of vesicles associated with clathrin-mediated endocytosis [reviewed in (30)]. Not much is known about the association of clathrin with glaucoma. However, northern blot analysis of gene expression patterns in PEX patients has shown an increase in clathrin expression in the lens epithelium (31). Generally, it has been discussed that uptake of circulating antibodies is conveyed by clathrin-mediated endocytosis that opens the opportunity of autoantibodies to cause various effects on an intracellular level (32, 33). Antibodies to clathrin, however, have not been reported in relation to PEXG. Circulating antibodies are hypothetically attributed to many different functions, including agonistic and antagonistic receptor effects or activation of the complement cascade (34). Stimulating as well as inhibitory effects are possible. The aggregation of exfoliation material in the anterior chamber has vision-threatening effects in PEX patients. Polymorphisms in the LOXL1 gene contribute to the accumulation of elastic myofibrils and extracellular matrix that impair the physiological function of the TM, leading to increased IOP and severe damage to the optic nerve as a consequence (35). Possibly, an altered clathrin-mediated extracellular matrix turnover in PEXG patients plays a role in the pathogenesis. Thus, the decreased autoantibody levels might be a consequence or precursor of this condition. Further studies are necessary to investigate the causalities of clathrin and anti-clathrin antibodies in PEXG.

Although perturbations in levels of serological antibodies might hint toward disease-related alterations in quality or quantity of the corresponding autoantigen, the conclusions one can draw of this remain vague. It is also not possible to deduce possible disease-related effects merely of the levels of single specific antibodies. Therefore, it is important to evaluate changes in the serological antibody repertoire not isolated, but in a holistic approach, using all available information. Thus, we took one step further and analyzed the entirety of known OAG-related autoantibodies to identify commonalities and biological processes that are the main crossroads for the immunological contribution in OAG. Autoantibodies to ocular antigens that show altered levels in glaucoma patients have already been described by numerous different studies conducted by various research groups. To learn more about the nature of the autoantigens identified in the past and in this present study, we implemented a holistic bioinformatics analysis to investigate the relations between these glaucoma antigens. We assumed that the examination of how they connect to each other in the context of the disease could help for a better understanding of synergies between autoimmune effects and other disease mechanisms. We found 13 biological processes enriched among the glaucoma-related antigens. Proteins were most highly enriched in regulation of mRNA processes, followed by protein folding, blood coagulation and apoptosis. Pathological changes in these processes were found in OAG patients and we assume a glaucoma—related association between alterations of these processes and corresponding autoantibody levels. Deregulations of RNA metabolism associated processes are a common feature of neurodegenerative diseases (36). Alterations in the regulation of some glaucoma-related mRNAs were also reported in TM cells of glaucoma patients (37), showing a possible relation to altered autoantibody abundance. The enrichment of antigens that are part of the biological process of protein folding arises mainly from overrepresentation of molecular chaperons. Disturbed protein folding can cause formation of protein aggregates that, when accumulating, can induce the unfolded protein response (UPR), which protects the cell from endoplasmic reticulum (ER) stress (38). ER stress in turn can lead to apoptosis if the capacities of the UPR are exceeded. ER stress—induced apoptosis is common in neurodegenerative diseases and also occurs in glaucoma pathogenesis (39). Abnormal blood coagulation was described in POAG patients in form of age-dependent spontaneous platelet aggregation (40) and platelet activation was also assumed to be involved in glaucoma pathomechanisms (41). Glaucoma-related autoantibodies were shown to especially reflect the platelet-derived growth factor receptor pathway (16). Whether the detected autoantibodies partake in the deregulation of these processes or act as possible countermeasures cannot be answered here. We can however assume a strong influence of these alterations on the serological autoantibody repertoire, presumably reflecting the antigenic status.

Findings from the PPI network suggest a special role in glaucoma associated immunity for the six antigens clustered by the MCODE algorithm. These antigens are mainly associated with mitochondria and apoptotic mitochondrial changes. In general, apoptosis is considered the main mechanism of RGC demise in glaucoma (42) and previous studies already emphasized the importance of mitochondrial dysfunction as an aspect in glaucoma pathogenesis, which is also considered a major cause of cell death of RGCs (43, 44). Pro-apoptotic alterations in the mitochondrial proteome have already been observed in retinal cells after incubation with serum of POAG patients (45). This indicates that serological autoantibodies are capable to promote disease progression and persistent neuronal damage. Future work should further investigate the connection and molecular mechanisms of a possible antibody-mediated impairment of mitochondria in RGCs.

Additionally, four of the six MCODE clustered antigens are also located in myelin sheaths. Conventionally, glaucoma is not considered as a demyelinating disorder. Recent investigations however found evidence that demyelination could play a role in the mechanisms causing neuronal damage (46). Although there is some inconsistency in data, suggesting that this could not be true for all cases (47). Additionally, a study in DBA/2J mice has shown that early insults of the optic nerve occur in proximity to the lamina cribrosa and that axon segments in the lamina and more distal undergo degeneration (48). Retinal ganglion cells are unmyelinated until they pass through the lamina cribrosa, but the myelinated axons beyond might be targeted by glaucoma-related antibodies. Autoantibodies to proteins of the myelin sheath are also known form other diseases such as multiple sclerosis and neuromyelitis optica. Here, antibodies against myelin-associated proteins [e.g., anti-myelin oligodendrocyte glycoprotein, anti-myelin basic protein (49) and anti-aquaporin 4] are frequently found in patients. Their role in the pathogenesis is not finally clarified yet. Though it is assumed that they have the potential to inflict axonal damage and inflammatory demyelination, which would also apply to the optic nerve cells distal to the lamina cribrosa (49, 50). This gives rise to the hypothesis that damages of the optic nerve sheath mediated by antibodies could also be capable to cause neuronal damage in glaucoma. On the other hand, IgG autoantibodies have been described to be involved in the debris clearance after injury of neuronal cells of the peripheral nerve system, thereby supporting axon regeneration (51). However, regardless of the effect of the IgG class autoantibodies, their occurrence is strongly linked to injuries of nerve cells and their axons. The antibody-mediated effects affecting glaucoma pathogenesis have not yet been elucidated and will require further investigation in future studies.

While our study gives new insights into autoantibodies in glaucoma, it also has some limitations that should be acknowledged. The detection of decreased levels of CLTA/B/C antibodies in serum of PEXG patients was conducted by protein microarrays as sole method. A validation with additional methods would increase the significance of this discovery. Also, more experiments are needed to elucidate the biological mechanisms behind this finding. This monocentric study is further limited by its relatively small sample size and unequal group size, which we attempted to deal with by using significance tests for unequal *N*. Furthermore, it will be interesting to see, whether patients suffering from pseudoexfoliation syndrome without glaucoma also exhibit lower levels of clathrin antibodies. Nevertheless, this marker can be useful to distinguish PEXG from other forms of glaucoma.

Overall, these results contribute to the assumption that serological autoantibodies can reflect pathological changes in the affected sites of the eye. Glaucoma-related autoantigens are enriched in biological processes with a strong link to previously described pathomechanisms. The causality of autoantibody genesis and corresponding pathological events, however, still remains a conundrum. Even though the molecular mechanisms of autoantibodies in glaucoma cannot be revealed in our study, it delivers enough evidence to justify future studies investigating the interplay of the immune system and glaucoma in the here identified biological processes in more detail. Apart from that, the observation of IgG level alterations in serum can be exploited for the monitoring of the disease and usage for diagnostic purposes, nonetheless. The detection of significantly altered autoantibody levels to clathrin promotes prospective diagnostic glaucoma-subtyping, especially for the discrimination of PEXG from other forms of OAG.

## Materials and Methods

### Sera

Sample collection was carried out in accordance with the Declaration of Helsinki on biomedical research involving human subjects. Written informed consent was obtained from each subject. The study was approved by the ethics committee of the Landesärztekammer Rheinland-Pfalz. All subjects included in this study received an ophthalmic examination at the department of ophthalmology of the university medical center in Mainz, Germany. Glaucoma patients were diagnosed according to the guidelines of the European Glaucoma Society (52). Visual field defects were determined using OCTOPUS 101 Perimeter (Haag-Streit, Wedel, Germany) or Humphrey Visual Field analyzer (Carl Zeiss Meditec, Dublin, CA). Correlation analysis was only run with OCTOPUS data sets, to avoid systematic bias. The control group consists of non-glaucomatous subjects. According to the anamnestic query, none of the included subjects suffered from Alzheimer's or Parkinson's disease. One subject of the non-glaucomatous control group reported suffering from multiple sclerosis. Glaucoma patients were diagnosed based on glaucomatous optic disc damage and typical visual field defects. All patients had open iridiocorneal angles. PEXG was diagnosed when patients showed PEX material in at least on eye. PEXG and POAG patients showed an IOP >21 mmHg, while IOP in NTG patients was <21 mmHg. The demographics of the study population can be found in Table 5.

**Table 5:** Demographics of study population.

	***N***	**m/f**	**Age (min–max)**
CTRL	46	27/19	66.96 (34–83)
NTG	31	15/16	68.29 (49–80)
PEXG	45	21/24	70.47 (52–86)
POAG	43	25/18	66.37 (24–81)

### Antigen Microarray Analysis

Recombinant or purified proteins selected for the antigen microarray are listed in Supplementary File 1. The arrays were produced in our lab, as described elsewhere (14). Array hybridization was performed using 16-well incubation chambers (ProPlate Multiwell chambers, Grace Biolabs, Bend, USA). All incubation steps were carried out on an orbital shaker at 4°C. Arrays were incubated for 1 h with a blocking buffer (Super G, Grace Biolabs, Bend, Oregon, USA). Then, the blocking buffer was removed, and the slides were washed three times with phosphate-buffered saline containing 0.5% Tween-20 (PBST). Subsequently, the arrays were incubated with 100 μL serum in a 1:250 in PBS overnight. As negative control PBS only arrays were included on each slide. Next, slides were washed three times again with PBST followed by incubation with an anti-human antibody conjugated with a fluorophore (Alexa Fluor® 647 AffiniPure Goat Anti-Human IgG, Fcγ fragment specific, 109-605-008, Jackson Immunoresearch) as secondary antibody diluted 1:500 in PBS for 1 h. Next, the arrays were washed twice with PBST and twice with ultrapure water. Lastly, the slides were dried for 2 min in a vacuum centrifuge concentrator (SpeedVac, Thermo Scientific, Waltham, Massachusetts, USA).

Array images were acquired as 16-bit TIF file using a high-resolution confocal laser scanner (428 Array Scanner, Affymetrix, Santa Clara, California, USA). The image analysis software Imagene (Imagene 5.5, BioDiscovery Inc., Los Angeles, California, USA) has been used to quantify spot intensities. Poor quality spots have been manually flagged and removed from the analysis.

### Microarray Data Pre-procession

Net signal intensities were calculated by subtraction of local background intensity. Signals reaching negative values after background subtraction were treated as missing data. Negative control signal intensities were subtracted from each spot. Intensities from the triplicate spots were averaged, yielding one mean fluorescence intensity. All signals were then normalized to the IgG control spots included on each subarray by median centring to reduce intra—slide variability and batch effects. Therefore, IgG median signal intensities were divided by the overall IgG signal median to obtain a factor for each subarray. All further analyses are based on these normalized fluorescence intensities (NFI). To ensure the robustness of the dataset and reduce the influence of outliers, values below the 5th and above the 95th percentile in each group were set as missing data. Only data above the defined limit of detection (mean fluorescence intensity from the negative control of each antigen + standard deviation) were eligible. Targets with overall more than 30% missing data were not eligible for statistical analyses. Missing data of targets with <30% missing values was imputed using the *k*-nearest-neighbor (KNN) algorithm. Grand mean normalization was applied for all arrays.

### Statistical Analysis

Statistical analyses were performed using Statistica (Statistica 13, Statsoft, Tulsa, Oklahoma, USA). One-way ANOVA with consecutive Tukey's HSD *post-hoc* test for unequal n was performed to determine significant alterations in antibody levels among the study groups. Pearson's correlation coefficients were used to evaluate correlations of autoantibody levels and clinical parameters. A *P*-value of < 0.05 was considered statistically significant. Heat mapping and k means clustering was implemented using Morpheus (https://software.broadinstitute.org/morpheus/).

### Protein-Protein Interaction Networks and Gene Ontology Enrichment Analysis

Gene Ontology enrichment analysis and the protein-protein interaction network was conducted in Metascape (http://metascape.org) (53). Also, Metascape's molecular complex detection (MCODE) algorithm was used to detect densely connected network components.

## Data Availability Statement

The raw data supporting the conclusions of this article will be made available by the authors, without undue reservation.

## Ethics Statement

The studies involving human participants were reviewed and approved by Ethics Committee of the Landesärztekammer Rheinland-Pfalz. The patients/participants provided their written informed consent to participate in this study.

## Author Contributions

VB planned the experimental design of this study, performed bioinformatics and microarray analysis, interpreted the data, and wrote the manuscript. FG critically revised the manuscript. FG and NP contributed to conception of the study and provided resources. All authors contributed to the article and approved the submitted version.

## Conflict of Interest

The authors declare that the research was conducted in the absence of any commercial or financial relationships that could be construed as a potential conflict of interest.
