# Supplementary material for: Serological Levels of Anti-clathrin Antibodies Are Decreased in Patients With Pseudoexfoliation Glaucoma
Source: Front Immunol. 2021 Feb 19;12:616421. doi: 10.3389/fimmu.2021.616421 (PMC7933590; doi:10.3389/fimmu.2021.616421)
Supplement: Supplementary file 1 [file Data_Sheet_1.docx]

| **Subtype-specific alterations in the serological autoantibody profile of open-angle glaucoma patients** |
| --- |
| Vanessa M. Beutgen, Norbert Pfeiffer, Franz H. Grus |

Supplementary Data

**S1 Supplementary file 1:** Recombinant and purified proteins on antigen microarray.

**Table S1 - 1** List of analysed antigens.

| **#** | **Gene name** | **Protein name** | **Supplier** |
| --- | --- | --- | --- |
| 1 | ACTA1 | Actin, bovine | Sigma-Aldrich |
| 2 | ACTN1 | Alpha-actinin-1 | abnova |
| 3 | ADRB2 | β-2 adrenergic receptor | Sigma-Aldrich |
| 4 | ANP32B | Acidic leucine-rich nuclear phosphoprotein 32 family member B | abnova |
| 5 | ATP5A1 | ATP synthase subunit alpha, mitochondrial | Aviva systems biology |
| 6 | C1QBP | Complement component 1 Q subcomponent-binding protein, mitochondrial | abnova |
| 7 | CALD1 | Caldesmon | Abcam |
| 8 | CCDC 42 | Coiled-coil domain-containing protein 42 | sino biological |
| 9 | CLTA/B/C | Clathrin, bovine | Sigma-Aldrich |
| 10 | COPA | Coatomer subunit alpha | abnova |
| 11 | CRYGS | Gamma-crystallin S | Abcam |
| 12 | DDX46 | Probable ATP-dependent RNA helicase DDX46 | creative biomart |
| 13 | ENO1 | Alpha-enolase | Abcam |
| 14 | FLNA | Filamin-A | abnova |
| 15 | FN1 | Fibronectin | Sigma-Aldrich |
| 16 | GFAP | Glial fibrillary acidic protein | US Biological |
| 17 | HNRNPF | Heterogeneous nuclear ribonucleoprotein F | abnova |
| 18 | HSP27 | Heat shock protein beta-1 | Enzo Life Science |
| 19 | HSP70 | Heat shock 70 kDa protein 1A | Abcam |
| 20 | HTRA2 | Serine protease HTRA2, mitochondrial | Novoprotein |
| 21 | IQGAP1 | Ras GTPase-activating-like protein IQGAP1 | abnova |
| 22 | MBP | Myelin basic protein, bovine | Sigma-Aldrich |
| 23 | MCM7 | DNA replication licensing factor MCM7 | abnova |
| 24 | MCTS1 | Malignant T-cell-amplified sequence 1 | abnova |
| 25 | MECP2 | Methyl-CpG-binding protein 2 | Novoprotein |
| 26 | MYO1C | Unconventional myosin-Ic | abnova |
| 27 | PKM2 | Pyruvate kinase PKM | Abcam |
| 28 | PNMA2 | Paraneoplastic antigen Ma2 | abnova |
| 29 | PPP2R2B | Serine/threonine-protein phosphatase 2A 55 kDa regulatory subunit B beta isoform | abnova |
| 30 | PROK | Proteinase K from *Tritirachium album* | Sigma-Aldrich |
| 31 | SERPINA1 | Alpha-1-antitrypsin | Sigma-Aldrich |
| 32 | SFN | 14-3-3 protein sigma | Enzo Life Science |
| 33 | SOD | Superoxide dismutase [Cu-Zn] | Sigma |
| 34 | TARS | Threonine--tRNA ligase 1, cytoplasmic | abnova |
| 35 | TGFB1I1 | Transforming growth factor beta-1-induced transcript 1 protein | Abnova |
| 36 | TTR | Transthyretin | Sigma-Aldrich |
| 37 | VDAC2 | Voltage-dependent anion-selective channel protein 2 | Abcam |
| 38 | VIM | Vimentin | ProGen |


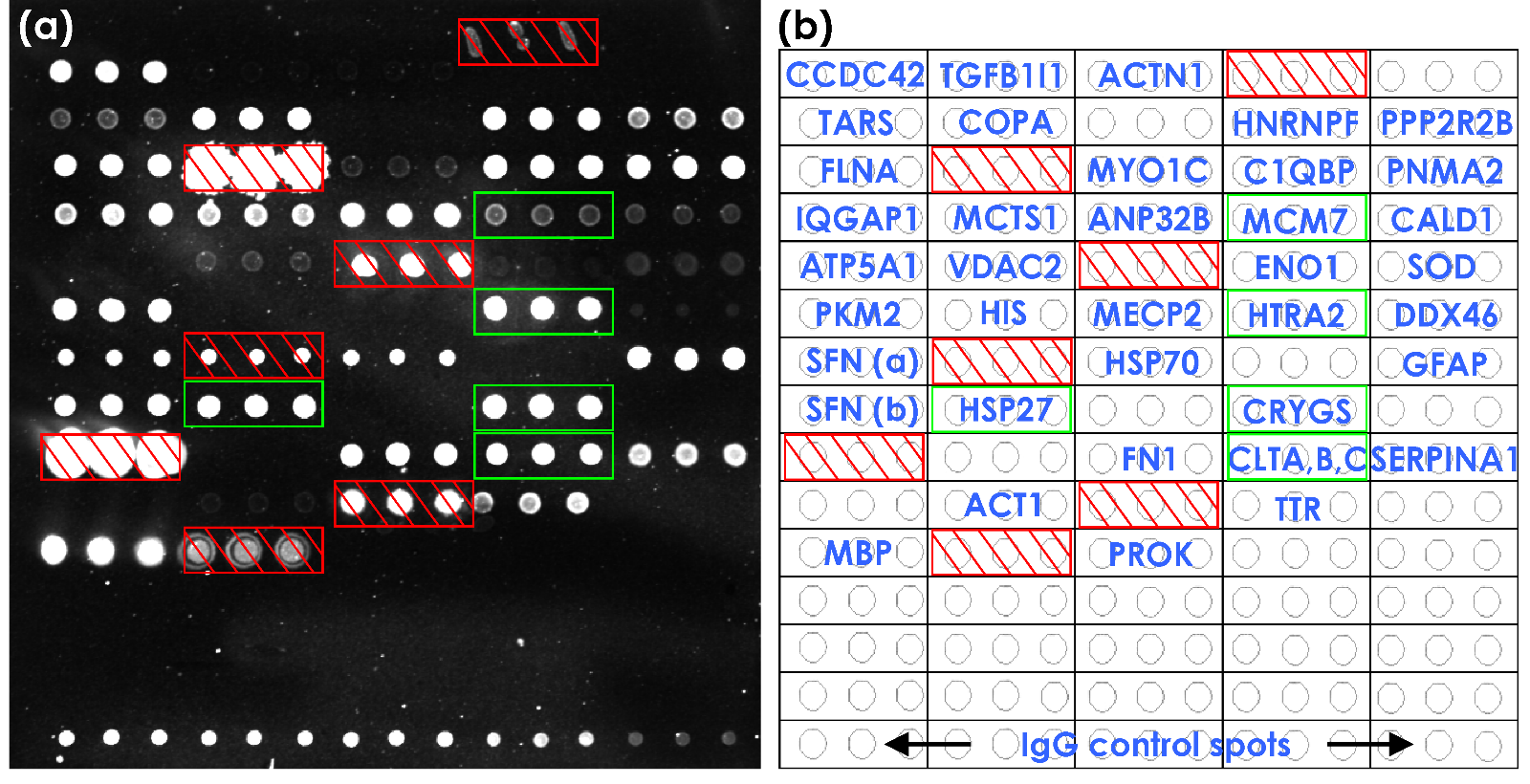


**Figure S1 - 1** Array design. **(a)** Example of scanned array. **(b)** Spot location of analysed antigens. Hatched red rectangles indicate defective spots that did not consistently meet the required quality criteria. Green rectangles indicate features with significant group differences as evaluated by ANOVA.
