# Supplementary material for: Serological Levels of Anti-clathrin Antibodies Are Decreased in Patients With Pseudoexfoliation Glaucoma
Source: Front Immunol. 2021 Feb 19;12:616421. doi: 10.3389/fimmu.2021.616421 (PMC7933590; doi:10.3389/fimmu.2021.616421)
Supplement: Supplementary file 2 [file Data_Sheet_2.docx]

| **Subtype-specific alterations in the serological autoantibody profile of open-angle glaucoma patients** |
| --- |
| Vanessa M. Beutgen, Norbert Pfeiffer, Franz H. Grus |

Supplementary Data

**S2 Supplementary file 2:** Post hoc test of significant ANOVA results. HSD test for unequal N was applied. Tables show respective P values.

**Table S2 - 1** ANOVA post hoc test for MCM7. HSD for unequal N.

| Group | Unequal N HSD; Variable: **MCM7** Marked (*) differences are significant at p < .05000 | | | |
| --- | --- | --- | --- | --- |
|  | \| {1} M=6.9070 \| \| --- \| | \| {2} M=7.1808 \| \| --- \| | \| {3} M=7.0444 \| \| --- \| | \| {4} M=7.4336 \| \| --- \| |
| \| NTG {1} \| \| --- \| |  | 0,544786 | 0,909707 | 0,051964 |
| \| CTRL {2} \| \| --- \| | 0,544786 |  | 0,855649 | 0,461392 |
| \| PEXG {3} \| \| --- \| | 0,909707 | 0,855649 |  | 0,110207 |
| \| POAG {4} \| \| --- \| | 0,051964 | 0,461392 | 0,110207 |  |

**Table S2 - 2** ANOVA post hoc test for HTRA2. HSD for unequal N.

| Group | Unequal N HSD; Variable: **HTRA2** Marked (*) differences are significant at p < .05000 | | | |
| --- | --- | --- | --- | --- |
|  | \| {1} M=9.7078 \| \| --- \| | \| {2} M=10.070 \| \| --- \| | \| {3} M=9.5585 \| \| --- \| | \| {4} M=9.8786 \| \| --- \| |
| \| NTG {1} \| \| --- \| |  | 0,333242 | 0,899597 | 0,857645 |
| \| CTRL {2} \| \| --- \| | 0,333242 |  | * 0,021893 | 0,714546 |
| \| PEXG {3} \| \| --- \| | 0,899597 | * 0,021893 |  | 0,287313 |
| \| POAG {4} \| \| --- \| | 0,857645 | 0,714546 | 0,287313 |  |

**Table S2 - 3** ANOVA post hoc test for HSP27. HSD for unequal N.

| Group | Unequal N HSD; Variable: **HSP27** Marked (*) differences are significant at p < .05000 | | | |
| --- | --- | --- | --- | --- |
|  | \| {1} M=12.015 \| \| --- \| | \| {2} M=11.856 \| \| --- \| | \| {3} M=12.369 \| \| --- \| | \| {4} M=11.870 \| \| --- \| |
| \| NTG {1} \| \| --- \| |  | 0,873940 | 0,326848 | 0,899867 |
| \| CTRL {2} \| \| --- \| | 0,873940 |  | * 0,016657 | 0,999844 |
| \| PEXG {3} \| \| --- \| | 0,326848 | * 0,016657 |  | * 0,023132 |
| \| POAG {4} \| \| --- \| | 0,899867 | 0,999844 | * 0,023132 |  |

**Table S2 - 4** ANOVA post hoc test for CRYGS. HSD for unequal N.

| Group | Unequal N HSD; Variable: **CRYGS** Marked (*) differences are significant at p < .05000 | | | |
| --- | --- | --- | --- | --- |
|  | \| {1} M=13.745 \| \| --- \| | \| {2} M=13.558 \| \| --- \| | \| {3} M=14.192 \| \| --- \| | \| {4} M=13.560 \| \| --- \| |
| \| NTG {1} \| \| --- \| |  | 0,789047 | 0,117142 | 0,794590 |
| \| CTRL {2} \| \| --- \| | 0,789047 |  | * 0,000860 | 0,999999 |
| \| PEXG {3} \| \| --- \| | 0,117142 | * 0,000860 |  | * 0,001061 |
| \| POAG {4} \| \| --- \| | 0,794590 | 0,999999 | * 0,001061 |  |

**Table S2 - 5** ANOVA post hoc test for CLTA/B/C. HSD for unequal N.

| Group | Unequal N HSD; Variable: **CLTA/B/C** Marked (*) differences are significant at p < .05000 | | | |
| --- | --- | --- | --- | --- |
|  | \| {1} M=11.166 \| \| --- \| | \| {2} M=10.891 \| \| --- \| | \| {3} M=8.6098 \| \| --- \| | \| {4} M=11.235 \| \| --- \| |
| \| NTG {1} \| \| --- \| |  | 0,933870 | * 0,000008 | 0,998844 |
| \| CTRL {2} \| \| --- \| | 0,933870 |  | * 0,000008 | 0,813021 |
| \| PEXG {3} \| \| --- \| | * 0,000008 | * 0,000008 |  | * 0,000008 |
| \| POAG {4} \| \| --- \| | 0,998844 | 0,813021 | * 0,000008 |  |


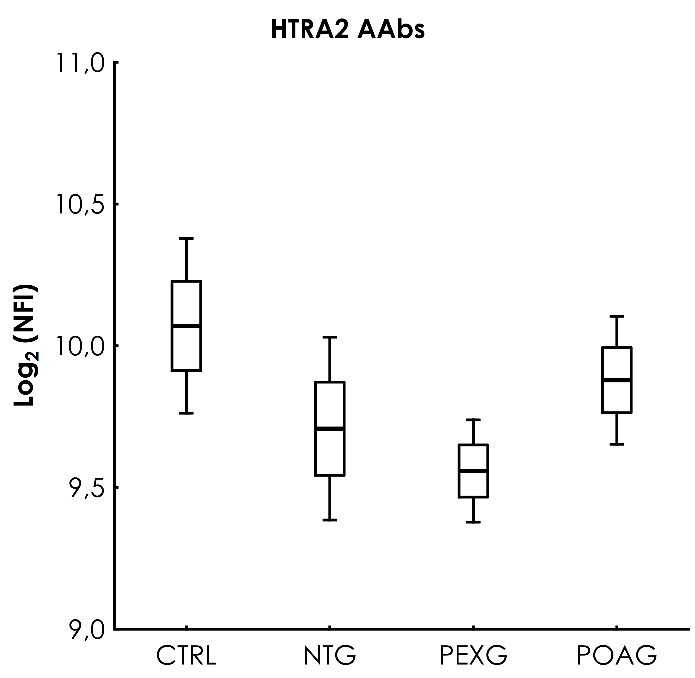


**Figure S2 - 1** Box plot of HTRA2 autoantibody levels in CTRL, NTG, PEXG & POAG.


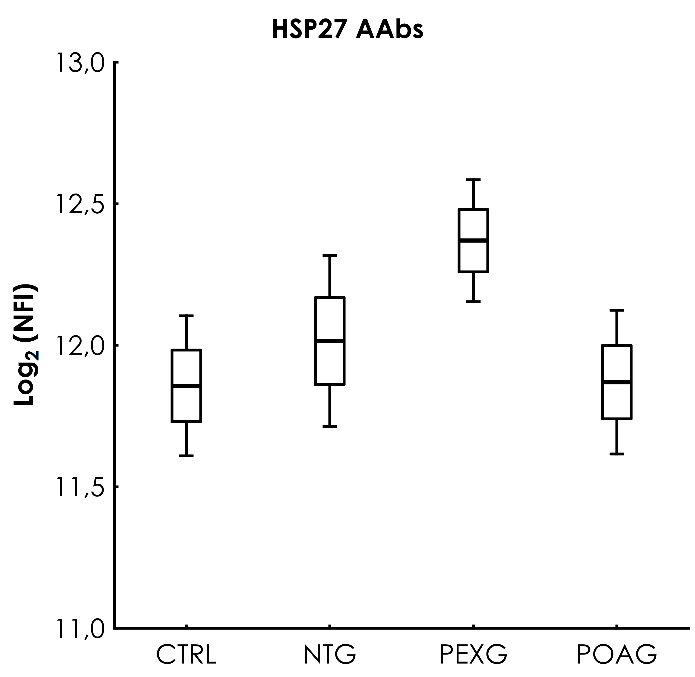


**Figure S2 - 2** Box plot of HSP27 autoantibody levels in CTRL, NTG, PEXG & POAG.


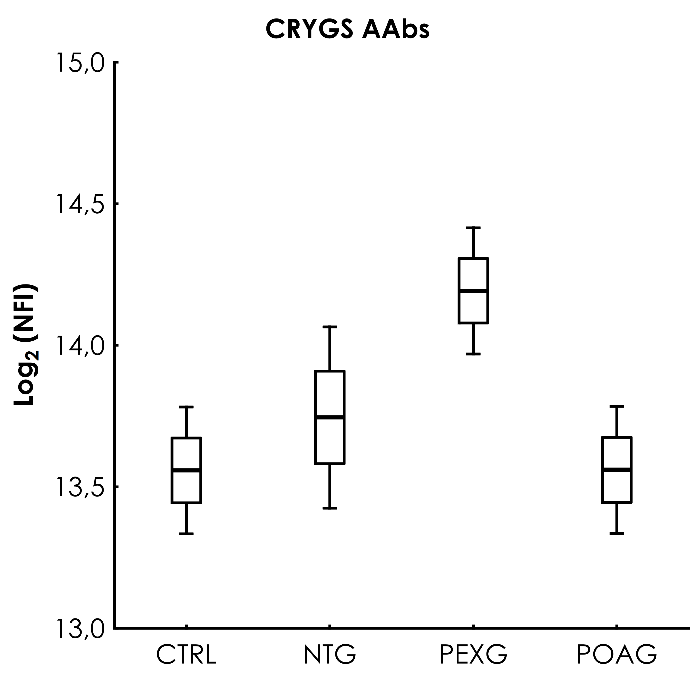


**Figure S2 - 3** Box plot of CRYGS autoantibody levels in CTRL, NTG, PEXG & POAG.
